# Efficacy of antimicrobial peptide P113 oral health care products on the reduction of oral bacteria number and dental plaque formation in a randomized clinical assessment

**DOI:** 10.1016/j.jds.2024.07.011

**Published:** 2024-07-19

**Authors:** Yi-Fan Wu, Bor-Cheng Han, Wen-Yi Lin, Sin-Yu Wang, Thu Ya Linn, Hsueh- Wen Hsu, Chih-Chieh Wen, Hung-Yi Liu, Yi-Hua Chen, Wei-Jen Chang

**Affiliations:** a School of Dentistry, College of Oral Medicine, Taipei Medical University, Taipei, Taiwan; b Department of Biomedical Engineering, Ming-Chuan University, Taoyuan, Taiwan; c School of Public Health, College of Public Health, Taipei Medical University, New Taipei City, Taiwan; d Neuroscience Research Center, Taipei Medical University, Taipei, Taiwan; e Research Center of Health Equity, College of Public Health, Taipei Medical University, New Taipei City, Taiwan; f Dental Department, Taipei Medical University, Shuang-Ho Hospital, New Taipei City, Taiwan

**Keywords:** Oral microbiota, P-113, Oral spray, PacBio SMRT sequencing, Dental plaque

## Abstract

**Background/purpose:**

Dental plaque is the main cause leading to the dental caries and periodontal diseases. The main purpose of this study was to test the efficacy of oral spray containing the antimicrobial peptide P-113 on the reduction of oral bacteria number and dental plaque formation in a randomized clinical assessment.

**Materials and methods:**

This study was divided into two parts. In Part A, we investigated the user experiences with the P-113 containing oral spray. In part B, 14 subjects in the experimental group used the P-113-containing oral spray, while 14 subjects in the control group used a placebo without the P-113 in a 4-week clinical trial. Participants were asked to use the P-113-containing oral spray or placebo 3 times per day and 5 times per use. Moreover, 3 check-ups and 2 washouts were carried out to evaluate the DMFT score, dental plaque weight, dental plaque index, and gingival index.

**Results:**

In part A, up to 91.8% of the subjects in the experimental group were satisfied with the use of the P-113-containing oral spray. In part B, based on our PacBio SMRT sequencing platform and DADA2 analysis, the numbers of *Streptococcus* and *Porphyromonas* in the experimental group were lower than those in the control group. In addition, decreased dental plaque weight, dental plaque index, and gingival index were all observed in the experimental group.

**Conclusion:**

The P-113-containing oral spray has the potential to reduce the dental caries and periodontal disease-related bacteria and to control the dental plaque formation.

## Introduction

Caries and periodontal diseases represent the most prevalent clinical diseases globally, affecting approximately 3.5 billion individuals and posing significant public health challenges.^1^ According to the report from the National Health Service, it is estimated that over 50% of the US adult population suffers from periodontal disease. Similarly, 53% of adults with periodontitis and 73% of elderlies with periodontitis in Taiwan.^2^ The most common oral diseases are bacterial in origin, with the oral cavity harboring over 700 bacterial species. This diverse microbiota includes anaerobic species like *Lactobacillus*, *Actinomyces*, *Veillonella*, *Porphyromonas*, and aerobic species such as *Pseudomonas, Streptococcus*.^3^

Many studies have shown that directly removing dental plaque biofilms from the tooth surface is effective for treating periodontitis.^4^ Oral cavity and periodontal pocket biofilms exhibit high resistance to antibiotic treatment. Recent reports also indicated that overuse of antibiotics has led to the emergence of drug-resistant bacterial strains.^5^ Traditional materials used in oral healthcare, such as metronidazole and chlorhexidine have been reported with some side effects. Thus, it's necessary to develop alternative methods for eliminating periodontal pathogens.^6^^,^^7^ Antimicrobial peptides known as AMPs are increasingly recognized as potent antimicrobial agents due to their rapid bactericidal properties and are also called host defense peptides.^8^ Currently, three models describe the mechanisms of action of AMPs, including the barrel-stave, toroidal pores, and carpet models.^9^^,^^10^ Histatins are small cationic histidine-rich peptides secreted by human parotid and submandibular glands with roles in forming the acquired enamel pellicle, inhibiting hemagglutination by periodontal pathogens, and exerting antimicrobial effects against bacteria and fungi to protect the oral cavity from pathogenic organisms.^11^^,^^12^ The characteristics of histatins like low toxicity and minimal immunogenicity, promote them as highly promising candidates for therapeutic and preventive applications in the oral cavity and other areas prone to localized infections.^13^^,^^14^ The efficacy of mouthwashes and mouth sprays in clinical settings is contingent upon the constituents comprising their formulations.^15^ P-113 is a 12-amino-acid peptide from histatin-5 with demonstrated efficacy against intracellular fungal targets and major microorganisms like *Streptococci*, *Staphylococci*, *Pseudomonas* spp., and *Candida albicans*.^16^^,^^17^ This suggests that oral care products with P-113 could be effective in managing oral microbial infections. However, some studies have indicated that the efficacy of P-113 diminishes greatly with high concentrations.^18^^,^^19^ A previous study involving 159 periodontally healthy subjects assessed the efficacy and safety of P-113 mouthrinse using eye drops after 28 days. Findings showed a notable decrease in dental plaque and plaque index for those using the 0.01% P-113 mouthrinse compared to the 0.005% P-113 and placebo groups.^20^ These findings suggest that P-113 mouthrinse, particularly at a concentration of 0.01% would reduce plaque and gingivitis without adverse effects effectively. The oral spray represents an efficacious approach to upholding oral health through the application of antiseptic agents.^21^ This method offers simplicity and ease of use, particularly beneficial for special needs individuals, including the geriatric population and those with physical or mental challenges. Furthermore, the targeted delivery inherent to the spraying technique enables precise treatment application, consequently minimizing drug dosage.^22^

This two-part, double-blinded, randomized clinical study was designed to evaluate the satisfaction and efficacy of a P-113-based oral healthcare product. The primary objectives were to assess user experiences and acceptance of the P-113 oral spray and to investigate its effectiveness in maintaining dental health and the potential to eliminate periodontal disease-causing oral bacteria. In Part A, we investigated user experiences with the P-113 containing oral spray through a questionnaire to understand the level of oral irritation and acceptance among consumers. In Part B, 28 participants were selected for an oral examination over a four-week treatment period, and 16S third-generation sequencing was utilized for oral microbiota analysis from dental plaque. This comprehensive approach combines subjective user evaluations with objective clinical measures to provide a thorough assessment of the P-113 oral spray's efficacy.

## Materials and methods

### Overall design

**Figure 1:**
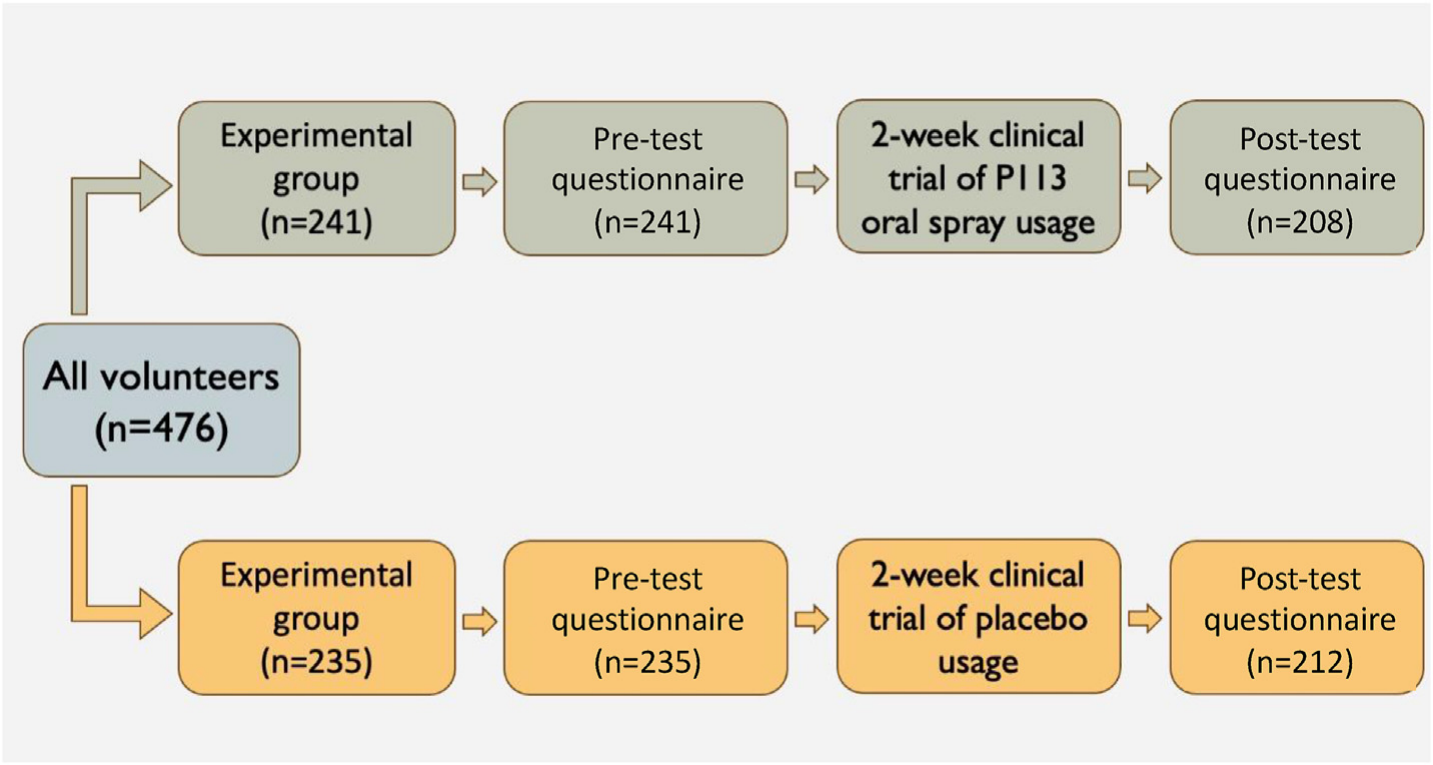
The flow chart of part A for the questionnaire's investigation.

The research received approval from the Institutional Review Board (IRB) at Taipei Medical University (Approval Number: N202203058). Comprising two distinct segments, the study encompassed **Part A** and Part B (See Fig. 1). In **Part A**, respondents were requested to complete pre- and post-usage questionnaires after applying of the P-113 containing oral spray, aiming to gauge customer satisfaction and product efficacy. Meanwhile, **Part B** involved the collection of dental plaque from volunteers for subsequent analysis. Statistical assessments encompassing changes in dental plaque, gingival index, plaque index, and the relative abundance of oral microbiota were conducted to ascertain the efficacy of the oral spray product in reducing periodontal pathogens and its potential to improve human oral health.

### Participants recruitment

Initially, corporate communications and social promotion recruitment information were disseminated within the corporate communication community by a university and hospital in northern Taiwan. Subsequently, participants voluntarily completed the study registration form on an individual basis. Then, the link to a pre-test electronic questionnaire, which included detailed information regarding the research process and consent form, was sent via email. Upon agreement, participants signed the consent form and returned it to the research team office prior to commencing the study. Secondly, health lectures were conducted, accompanied by the placement of recruitment posters outside the event venue. Interviewers explained the purpose and process of the study to the participants. The study commenced after participants agreed to participate and completed the consent form. Thirdly, further participant recruitment was facilitated by current participants referring study information to their relatives and friends. Prospective subjects willing to participate registered through an online registration form. Lastly, subjects from the hospital who met the study's inclusion criteria were recruited through the collaborative efforts of family medicine and dentistry departments from two teaching hospitals in Taipei City and New Taipei City. Subsequently, oral sprays with identical product appearance, color, and fragrance were delivered. Participants in the experimental group received P-113 oral spray, while those in the control group received a placebo.

### Study population and criteria

This study was conducted in two parts. In Part A, initially, 538 sheets were distributed, with 476 participants ultimately completing the clinical trial. These participants were subsequently divided into two cohorts: an experimental group (n = 241) and a control group (n = 235). Following the 14-day clinical trial period, a total of 420 participants, comprising 208 from the experimental group and 212 from the control group, completed the post-test questionnaire, representing a retention rate of 88.2%.

**Figure 2:**
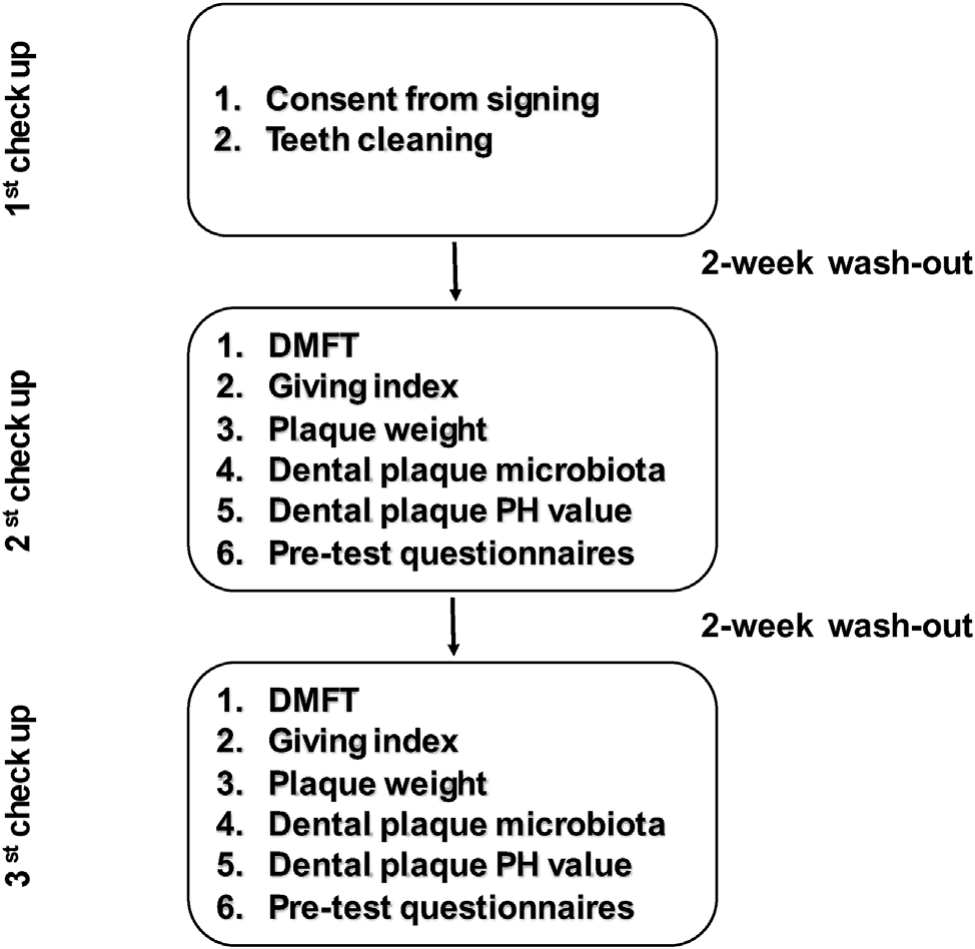
Flow chart of part B clinical trial. DMFT: sum of the number of decayed, missing, and filled teeth in the permanent teeth.

In Part B, a total of 28 volunteers were evenly allocated into two groups: an experimental group (n = 14) and a control group (n = 14). All volunteers, aged between 20 and 60 years, had prior dental conditions, including periodontitis, halitosis, exodontia, xerostomia, dental prosthetics, and dental orthodontic treatment. Exclusion criteria included pregnancy and severe systemic diseases. These volunteers were randomly allocated into an experimental group (n = 14) and a control group (n = 14) using stratified randomization to ensure balance in age and dental condition distribution between groups (see Fig. 2).

### Clinical trial procedure

In Part A, the questionnaires utilized were derived from the Geriatric Oral Health Assessment Index (GOHAI) through collaboration among four experts and the oral spray company. Prior to the commencement of the trial, participants were instructed to complete pre-test questionnaires. Subsequently, participants were randomly assigned to either the experimental or control groups for a double-blind, two-week clinical trial. The experimental group received an oral spray containing P-113, whereas the control group received a placebo lacking P-113. The placebo oral spray consisted of aqua, hydroxyethyl cellulose, sodium acetate trihydrate, saccharin sodium salt hydrate, disodium EDTA, 70% sorbitol solution, peppermint oil, PEG-40 hydrogenated castor oil, menthol crystals, and arginine. Both sprays were indistinguishable regarding exterior appearance, color, and taste. Following the trial period, participants were requested to complete the post-test questionnaire.

In Part B, a total of 28 participants were selected from both groups and subsequently enrolled in a 4-week clinical trial. Throughout the trial, three assessments were conducted. During the initial assessment, following consent, each participant underwent teeth cleaning procedures. Following a two-week washout period, the second assessment was performed, involving pre-evaluation such as the examination of the DMFT score, the gingival index score, plaque weight, and pH value of dental plaque. All 28 participants were randomly allocated into two groups: an experimental group (n = 14) and a control group (n = 14). The experimental group used 0.01% P-113 oral spray, while the control group used a placebo spray. Usage instructions were: apply thrice daily after meals, five sprays per use, avoiding eating or drinking for 30 min after application. Following a two-week treatment period, the third assessment was conducted for post-evaluation, which included examining the DMFT score, gingival index score, plaque weight, and pH value of dental plaque.

### Sample collection and metagenomic DNA extraction

Dental plaque samples from the supragingival area of the oral cavity were collected using a periodontal curette and promptly weighed. The same dentist conducted the entire sample collection process. Genomic DNA was then extracted from the plaque and prepared following the manufacturer's instructions outlined in the GenElute™ Bacterial Genomic DNA Kit (Sigma–Aldrich, St. Louis, MO, U.S.A). Each dental plaque sample underwent bacterial preparation according to the manufacturer's protocol. The resulting samples were placed into collection tubes containing elution solution (10 mM Tris–HCl, 0.5 mM EDTA, pH 9.0) for subsequent 16S amplicon sequencing. Subsequently, the concentrations of the extracted DNA were determined using a Nanodrop 2000 spectrophotometer (Thermo Fisher Scientific, Waltham, MA, U.S.A) and stored at −20 °C until further analysis.

### Microbiome library construction and sequencing

The bacterial community diversity within dental plaque was assessed using SMRT PacBio sequencing technology (Pacific Biosciences, Menlo Park, CA, U.S.A). The full-length 16S rDNA sequence was amplified according to the manufacturer's instructions, utilizing the bacterial-specific universal PCR primers 27F and 1492R. Following amplification, the final amplicon products were purified using AMPure PB beads (Agencourt, Beverly, Massachusetts, U.S.A) and quantified using a Qubit 2.0 Fluorometer with a Qubit® dsDNA HS Assay Kit (Thermo Fisher Scientific). DNA fragments were then fragmented, and adaptors were attached to both ends, rendering them detectable templates. A DNA sample library was constructed and subsequently amplified upon generating numerous templates, paving the way for the sequencing reaction.

### Statistical analysis

In Part A, McNemar's test was employed to examine whether there was an improvement in oral health issues following the application of the spray. In Part B, statistical analyses, such as the student's paired t-test and Wilcoxon rank sum test, were utilized to evaluate parameters such as plaque weight, plaque index, and gingival index. Additionally, correlation coefficients were computed using the Spearman test for other data.

## Results

### Part A. Thorough evaluation of P-113 oral spray usage

#### Effects of spray use—oral health issues

The impact of oral spray utilization on oral health issues (including gum bleeding, tooth or gum pain, dry mouth, bad breath, canker soreness and oral ulcers, etc.) was examined. Table 1 shows the outcomes observed in participants after using oral spray. Compared with the changes in oral health problem scores before use, the results showed that after using the spray, the oral health status scores of both the experimental group (*P* < 0.001) and the control group (*P* = 0.002) were significantly reduced, indicating that the oral health status has improved. The improvement in the experimental group was better than that in the control group, but there is no significant difference in the life scores of the oral quality between the two groups before and after using the spray (*P*-value ≥0.05).

**Table 1:** Pre and post-test changes in oral health problem scores (n = 420) *P*-values are the results of paired t-test testing, ∗*P* < 0.05. The score change is the post-test score minus the pre-test score. The higher the score, the worse the oral health status or the more serious impact the oral cavity has on the quality of life. Oral health conditions include, bleeding gums, tooth or gum pain, dry mouth, bad breath, and canker sore and mouth ulcers (Exp. Group: experimental group, Ctrl. Group: Control group).

Oral health status			
	mean	SD	*P*-values
Exp. group	−0.81	2.59	0.001∗
Ctrl. group	−0.56	2.54	0.002∗
Oral quality life scores			
	mean	SD	*P*-values
Exp. group	−0.53	4.69	0.11
Ctrl. group	−0.24	5.05	0.48

**Figure 3:**
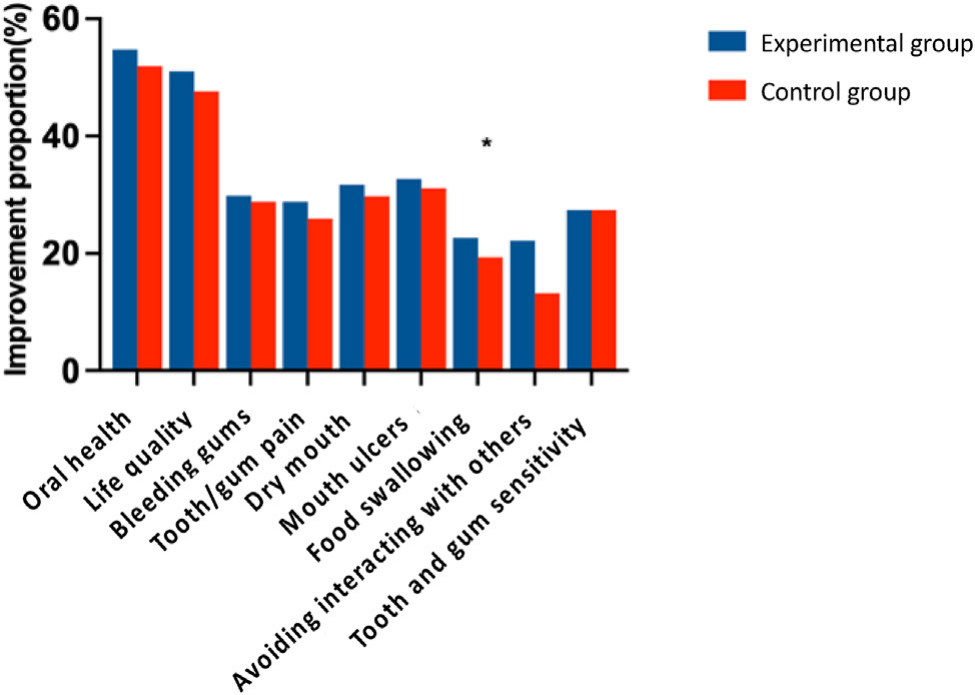
Improvement proportion of oral health problems. ∗*P* < 0.05.

Fig. 3 shows the improvement in oral health problems of participants after using the spray compared with before use among experiment and control groups. The results show that the improvement rate of food swallowing scores in the experimental group was significantly higher than in the control group (*P* = 0.02).

#### The spray usage experience of the experimental group - stratified analysis based on sub-groups

McNemar's tests were used to examine the proportion of severe oral health problems in the experimental group (spray containing P-113, n = 208) before and after using the spray. Compared to baseline, we found that the proportion of moderate to severe oral malodor in the experimental group significantly decreased from 35.0% to 27.7% after use (*P* = 0.03). Additionally, the proportion of moderate to severe dry mouth significantly decreased from 35.1% to 23.4% (*P* < 0.0001). Although not statistically significant, there were also trends toward improvement in the proportions of moderate to severe gum bleeding, tooth/gum sensitivity, and oral ulcers.

To further explore the overall formula of oral spray containing P-113ingredient in different socio-demographic groups, this section will explore the improvement effect of oral health problems of sub-groups after using the spray in the experimental group (spray containing P-113ingredient, n = 208), including bleeding gums, tooth and gum pain, dry mouth, bad breath, canker sore and oral ulcers, etc. The following analysis includes age (142 people “under 45 years old” and 66 people “over 45 years old”), gender (71 people “male” and 137 people “female”), oral health status (90 people with “poor” oral condition and “good with 118 people), teeth cleaning habits (“have” 121 people and “don't have” 87 people), frequency of spray use (“Use more than 3 times a day” 72 people and “Use less than 2 times a day” 136 people) is presented, and in addition, the experience of cancer patients (n = 12) is presented.

**Figure 4:**
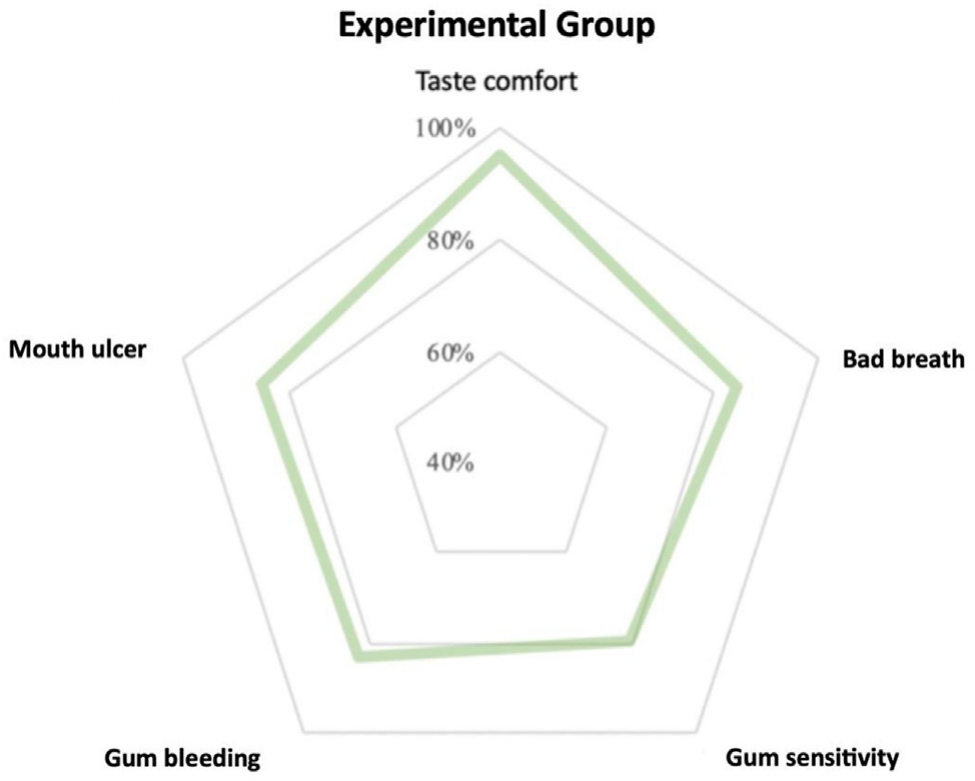
Positive feelings (maintained or better) of the experimental group.

First, Fig. 4 shows the proportion of positive feelings (maintained or better) of the experimental group (n = 208) after using the spray. The results show that after use, most participants found the spray tasted comfortable (95.1%). They had positive feelings about oral ulcers (85.1%), bad breath (84.6%), bleeding gums (83.2%), and tooth or gum sensitivity (79.8%).

**Figure 5:**
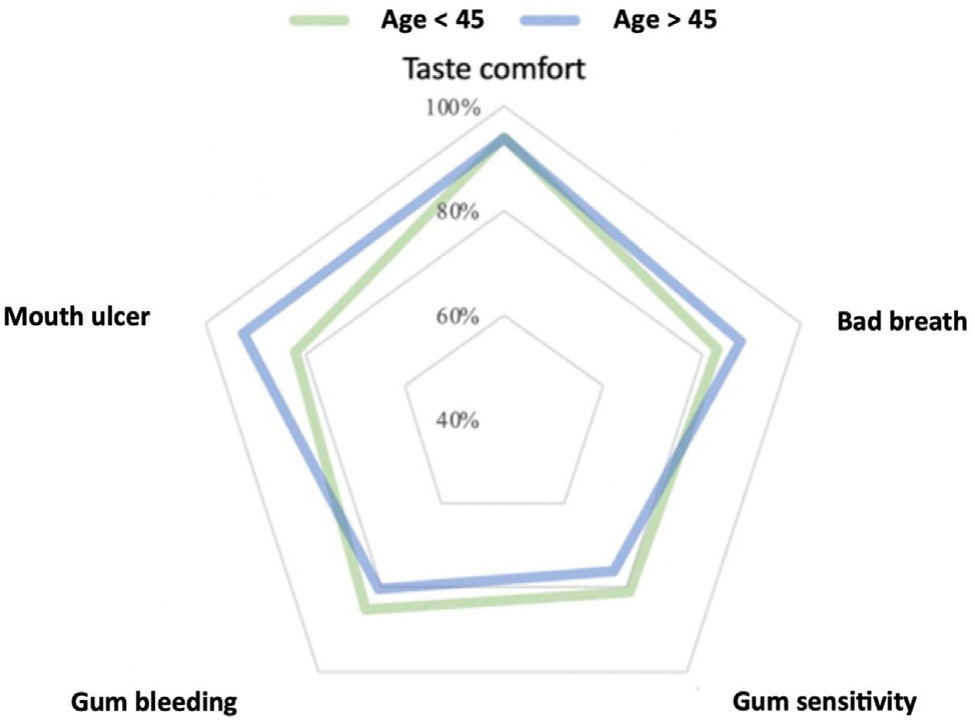
Positive feelings (maintained or better) based on age.

The results of age-stratified analysis showed that those younger than 45 years old (n = 142) were more likely to experience bleeding gums. The proportion of positive feelings about gum sensitivity (maintained or better) was slightly higher among those aged <45, while the proportion of positive feelings about oral ulcers and bad breath was higher among those over 45 years old (n = 66) shown in Fig. 5.

**Figure 6:**
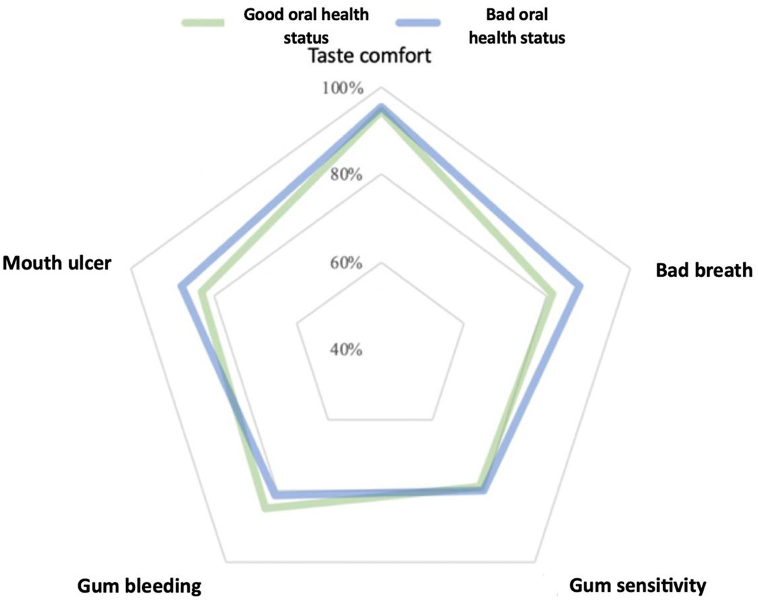
Positive feelings (maintained or better) based on oral health status.

Stratified analysis based on oral condition showed that the group with poor oral condition (n = 90) had oral ulcers. For issues such as ulcers and bad breath, the proportion of individuals reporting positive outcomes (either maintained or improved) following the use of the spray is notably higher, as shown in Fig. 6.

#### Oral spray usage experience and purchase intention

This section presents participants’ purposes for utilizing oral sprays and their subsequent inclinations toward purchasing them. The results indicate that a significant proportion of participants expressed satisfaction with the efficacy of oral sprays (92.1%) and believed that such oral sprays were beneficial for maintaining oral health (96.2%). Among the various purposes cited by participants for using oral sprays, the foremost objective was to ensure oral freshness (88.3%), followed by the desire to prevent periodontal disease (34.1%), tooth decay (28.8%), and to maintain oral moisture (22.5%). Furthermore, most participants were willing to purchase oral sprays (71.1%), with a substantial portion indicating a preference for expenditure within the range of 101–300 NT dollars (66.5%). When contemplating a purchase, participants predominantly prioritized efficacy (81.1%) as the most crucial factor, followed by considerations of price (63.2%), taste (33.9%), brand (15.0%), and outer packaging (2.9%).

### Part B. Analyzation of oral microbiome relative abundance and dental plaque

#### Dental plaque score and weight

**Figure 7:**
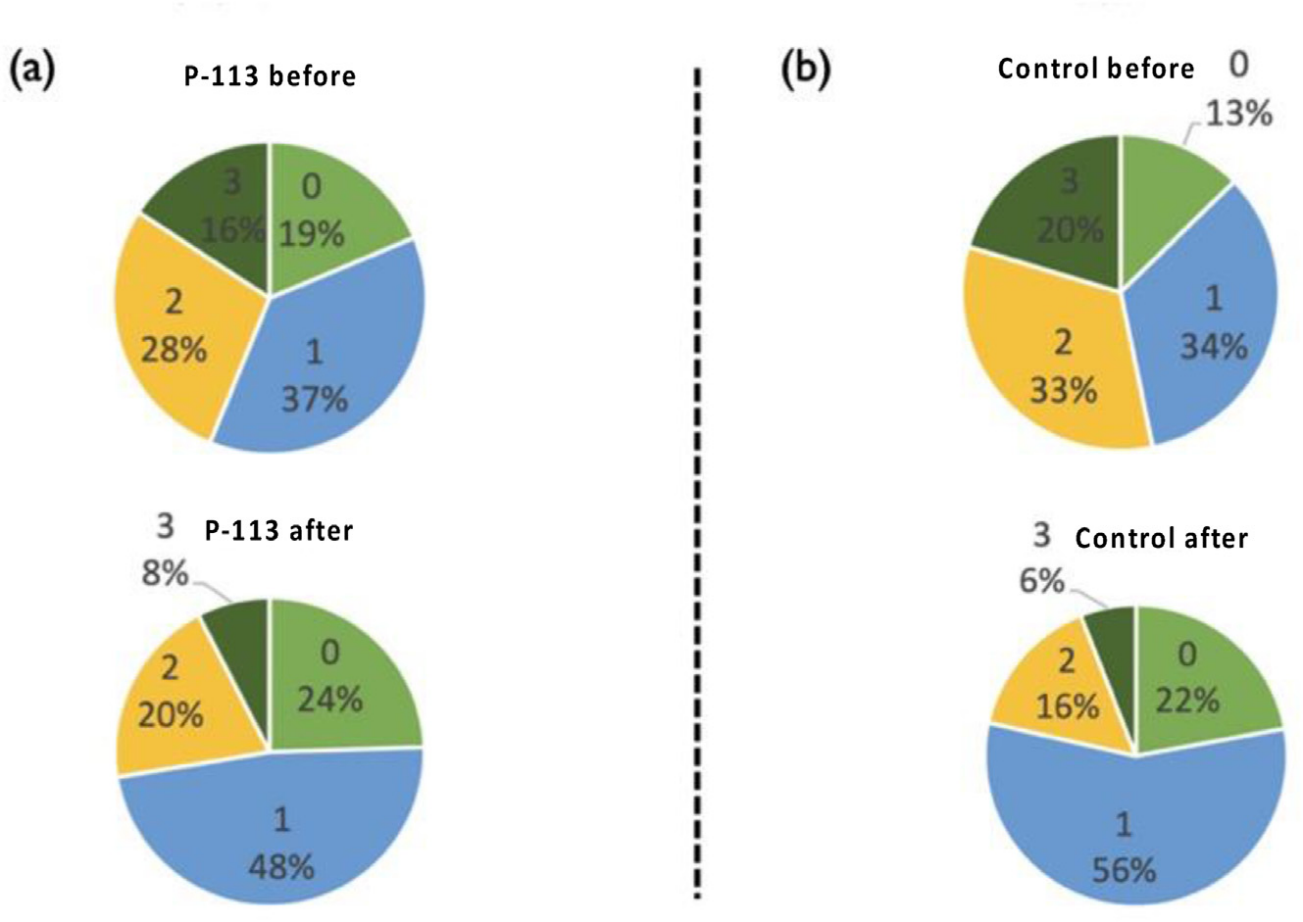
**(a)** Changes of dental plaque scores in the experimental group. **(b)** Changes of dental plaque scores in the control group.

Fig. 7 clearly shows improvement in participants' plaque scores regardless of whether they utilized oral sprays containing P-113 or not for both the experimental and control groups, particularly the grade 2 and grade 3 categories exhibiting a significant improvement. Regarding dental plaque weight, 10 participants in the experimental group demonstrated a decrease (median decrease of 6.3 mg), compared to only 7 participants in the control group (median rise of 3.3 mg).

#### Gingival index

**Figure 8:**
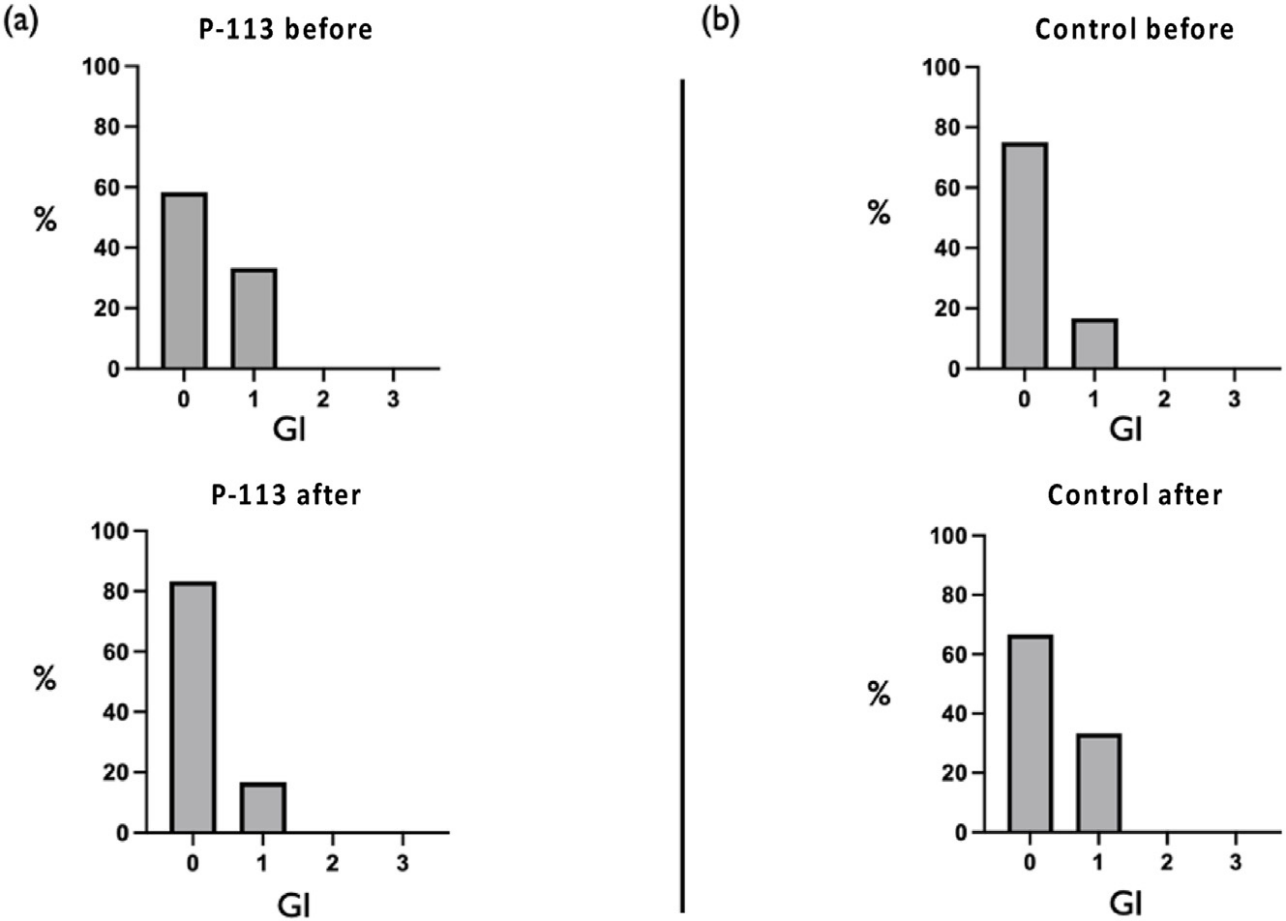
(a) Percentage changes of gingival index in the experimental group. (b) Percentage changes of gingival index scores in the control group. GI: Gingival index.

As shown in Fig. 8, the gingival index of 7 participants in the experimental group decreased, whereas in the control group, only 4 participants exhibited a reduction in their gingival index.

#### Oral microbiome examination

**Figure 9:**
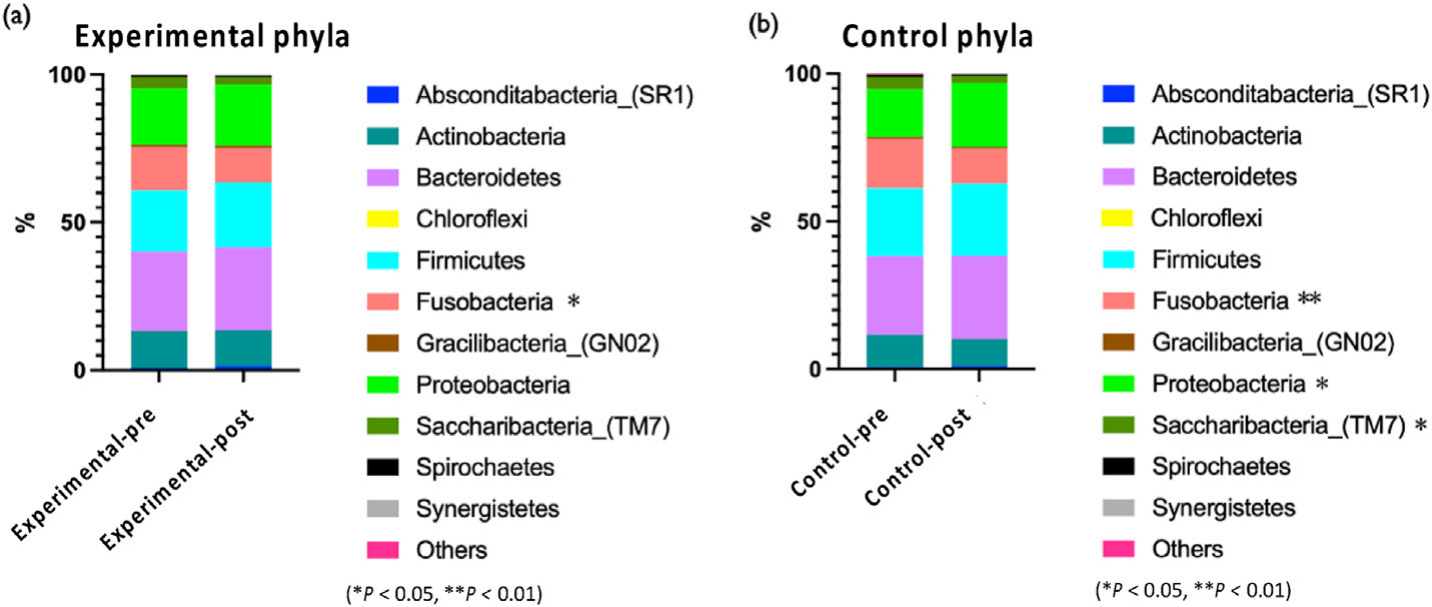
(a) Relative average abundance at the phlyum level from the experimental group. (b) Relative average abundance at the phylum level from the control group. ∗*P* < 0.05 and ∗∗*P* < 0.01, respectively.

Fig. 9 displays relative abundance at the phylum level. In the experimental group, *Fusobacteria* decreased from the original 14.70%–11.66%, the change decreased by 3.1% (−3.1%), with a significant difference (*P* < 0.05), while in the control group, *Fusobacteria* decreased from the original 16.70%–11.80%, the change decreased 4.9% (−4.9%), with significant difference (*P* < 0.01). *Bacteroidetes* in both the experimental and control groups all increased, with a rise of 1.3% and 1.4%, respectively. Same tread as *Proteobacteria*, statistics elevated in both groups. In the experimental group, *Proteobacteria* increased from the original 19.10%–20.70%, the change increased by 1.6% (+1.6%), but with no significant difference; while in the control group, *Proteobacteria* increased from the original 16.10%–21.70%, the change increased by 5.6% (+5.6%), with a significant difference (*P* < 0.05).

**Figure 10:**
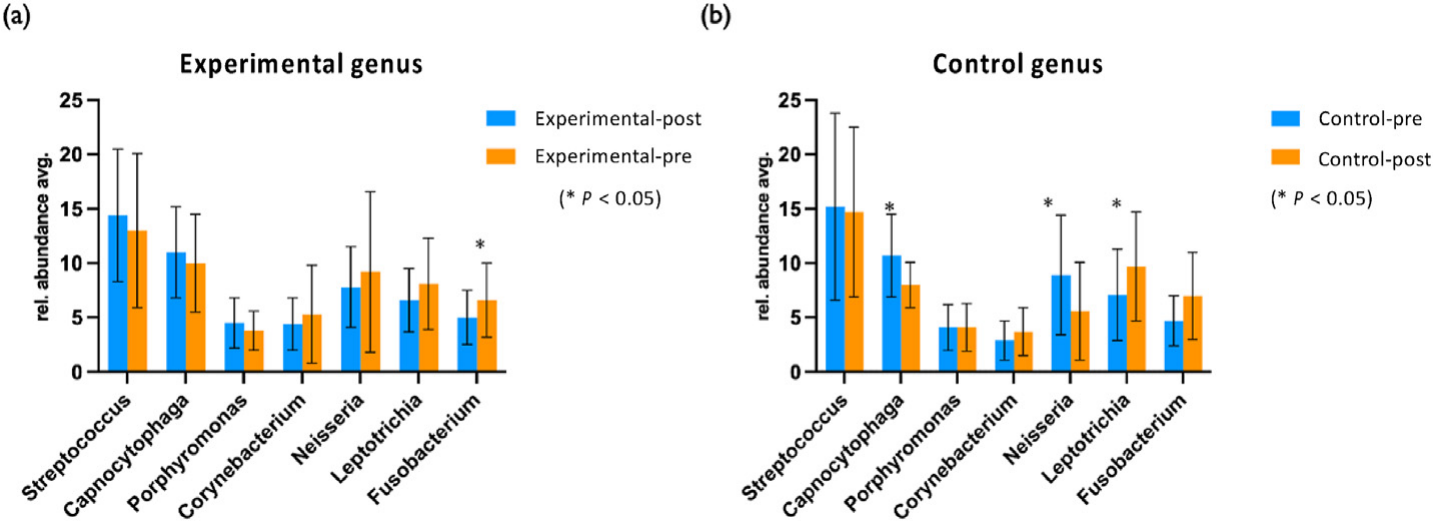
(a) Relative average abundance at the genus level from the experimental group. (b) Relative average abundance at the genus level from the control group. ∗*P* < 0.05.

The top 7 taxa at the genus level are shown in Fig. 10. After using P-113 oral spray, tooth caries-related bacteria such as *Streptococcus* in the experimental group dropped from 14.4% to 13.0%, with a change of 1.4% (−1.4%), while the control group only dropped from 15.2% to 14.7%, with a change of only 0.4% (−0.4%). It can be stated that using P-113 oral spray can help reduce the growth of tooth caries-related bacteria. *Porphyromonas* is a periodontal-related bacteria, decreased in the experimental group after using P-113 oral spray, from the original 4.55%–3.80%, the change decreased by 0.75% (−0.75%). In the control group, *Porphyromonas* increased from 4.11% to 4.14%, the change increased by 0.03% (+0.03%), indicating that using P-113 oral spray will help reduce periodontal pathogenic bacteria growth.

**Figure 11:**
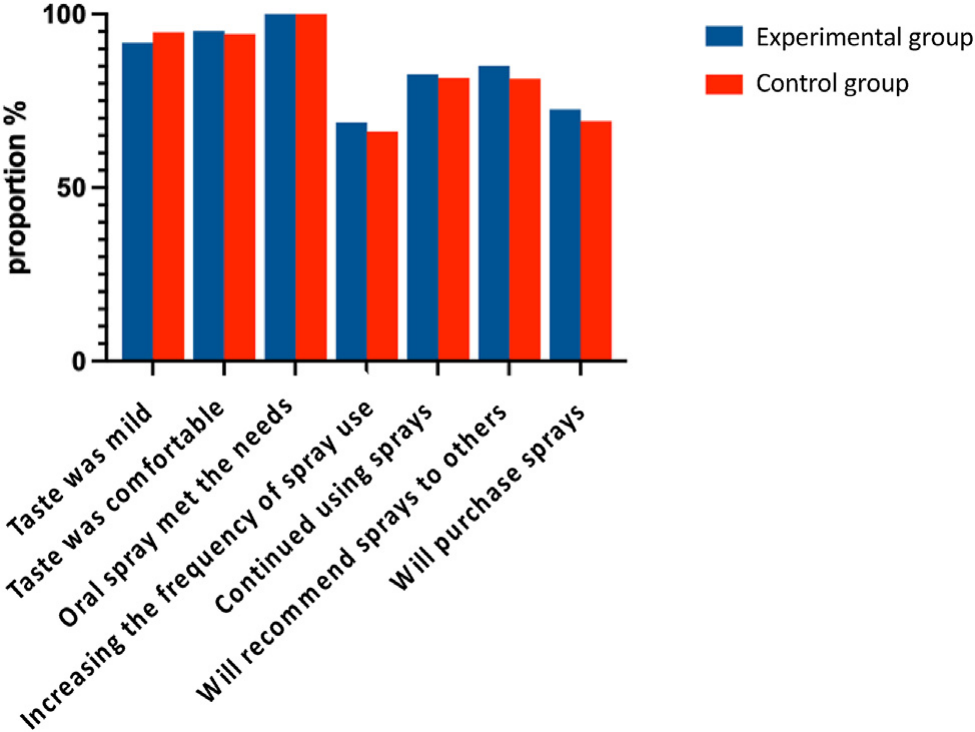
Effects of oral spray use-experience.

Finally, we also analyzed the effect of using oral spray on use experience, including taste, comfort, subsequent use recommendation intention, etc. Fig. 11 presents the participants’ feelings and experiences after using the spray.

## Discussion

In this comprehensive study, we aimed to investigate the effectiveness of P-113 oral spray in reducing dental plaque weight and formation, as well as its ability to decrease the accumulation of periodontal and caries-related bacteria. As in our previous studies,^23^^,^^24^ we employed the 16S rDNA PacBio SMRT sequencing technique to evaluate the relative abundance of the oral microbiome. Using the PacBio sequencing platform (PacBio SMRT; Pacific Biosciences of California, Inc., Menlo Park, CA, USA), this third-generation sequencing technology offers several advantages over previous short-read sequencing methods. It generates reads longer than 10 kb, enabling more accurate results and improved resolution of operational taxonomic units (OTUs).^25^ Additionally, it allows for identifying pathogenic variants from the V1–V9 hypervariable regions, with an average error rate lower than 1% by single-molecule consensus sequence.^26^ According to our results, *Streptococcus* and *Porphyromonas* at the genus level have dropped considerably in the experimental group (−1.4%) compared to the control group (+0.03%), although the difference between the two groups was not statistically significant. A previous study pointed out that amino-acid peptide P-113 has been proven to be effective in inhibiting periodontal bacteria such as *Streptococcus gordonii* and *Porphyromonas gingivalis* which are regarded as the early and late colonizers respectively of the bacterial aggregation in dental plaque biofilms.^27^ Therefore, we confirmed that P-113 has the potential to impede periodontal bacteria.

The questionnaire evaluation from Part A of the study revealed that both the experimental group (spray containing P-113) and the control group showed significant improvements in scores related to oral health problems, including gum bleeding, tooth/gum pain, dry mouth, oral malodor, and oral ulcers, from pre-test to post-test. However, the experimental group exhibited greater improvement than the control group, with a significantly higher proportion of improvement in swallowing food. The experimental spray contained the added ingredient P-113, a salivary antimicrobial peptide (histatin 5) that has been shown to possess anti-inflammatory properties and inhibit dental plaque, gingivitis, and gum bleeding.^28^ Given these properties, including P-113 in the experimental spray may be associated with the greater degree of improvement observed in the experimental group. Regarding the impact on oral health-related quality of life, as assessed by the questionnaire, although both groups showed improvements from pre-test to post-test, these improvements were not statistically significant. This lack of significance may be attributed to the subjective nature of quality of life, as participants might have found it easier to perceive direct improvements in oral health issues and gum conditions after using the spray rather than changes in their overall quality of life. Interestingly, the study observed that participants in the experimental group experienced significant improvements in tooth sensitivity after using the spray, while no significant improvements were noted in the control group. This finding suggests that P-113 may help reduce bacterial-induced immune responses, thereby controlling inflammatory reactions in the oral cavity.

Dental plaque biofilms are recognized as the direct cause of periodontal diseases and dental caries.^29^ Bacteria like *Streptococcus sanguis*, *Streptococcus. oralis*, and *Neisseria* sp., known as the early colonizers of the pellicle, grow and form the early plaque. These bacteria then serve as substrates for attachments of later colonizers like *Streptococcus mutans*, well known as the major cause of dental caries.^30^ In our study, *Streptococcus* sp. was restrained more significantly in the experimental group, but with no significant difference. Another study stated that P-113 mouthrinse is safe, can decrease dental plaque formation, and also reduces gingival bleeding and gingivitis in humans.^20^ In the present study, 10 participants in the experimental group showed a decrease in dental plaque (median decreased by 6.3 mg), while only 7 participants (median increased by 3.3 mg) in the control group showed a decrease. These changes demonstrated that P-113 can inhibit dental plaque formation, which is important in preventing both periodontal diseases and dental caries.

Notably, the *Capnocytophaga* genus in our results was reduced to a greater extent in the control group. Species like *C. gingivitis* was associated with gingivitis,^31^ and *C. ochracea* was found to be a beneficial species.^32^ Even though *Capnocytophaga* species may be associated with periodontal diseases, they still showed higher levels in health-associated individuals. We hypothesized that one of the reasons was that the pathogenicity among *Capnocytophaga* species was low, and due to the irregular placement, the pathogenicity may differ between species.^33^ Thus, *Capnocytophaga* can be found in both healthy and disease-related individuals. At the same time, the health-associated group presents the most, which is equivalent to our results with the higher percentage of *Capnocytophaga* in our experimental group.

Our randomized, double-blind design ensures the comparability of the two groups of subjects and improves the reliability and validity of the research results. This study analyzed the demographic variables, oral health status, long-term health problems, and behaviors of the two groups and found that there were no significant differences. The randomization process indeed achieved the expected result. Furthermore, the questionnaire designed in this study has good reliability and validity, and the overall response rate of the questionnaire is high, with a response rate of 88.5% and a tracking completion rate of 86.3% for the experimental group and 90.2% for the control group, which minimizes response bias. However, there are still some limitations in the present study that should be addressed in future studies. First, the PacBio platform requires a much higher cost. Lowering the cost could make further tests more accessible. Second, recruitment methods could be diversified. Third, the subjects we collected in Part B had a lower average age, which may limit the generalizability of the results. The subjects of this study were recruited from the corporate communication groups of a university and hospital. They generally have relatively high academic qualifications. Therefore, the effect of healthy subjects cannot be ruled out, and there might be a possibility of underestimating it. Fourth, our clinical trial involved only 28 participants (14 per group) over a 14-day period. This relatively small sample size and short duration may limit the statistical power and the ability to detect long-term effects or rare adverse events.

In conclusion, our study demonstrates that the P-113 oral spray significantly improves oral health. Key benefits include high user satisfaction (91.8%), reduced dental plaque, improved gingival health, and effective inhibition of oral pathogens like *Streptococcus* and *Porphyromonas*. These improvements, observed after just 14 days of use, along with the spray's ease of application, suggest that P-113 oral spray is a promising, user-friendly addition to daily oral hygiene solutions. However, longer-term studies with larger, diverse populations are needed to fully establish its efficacy.

## Declaration of competing interest

The authors have declared that there are no competing interests.
